# Low-Grade Appendiceal Mucinous Neoplasm Adhered to the Posterior Cecum: A Case Report

**DOI:** 10.7759/cureus.67313

**Published:** 2024-08-20

**Authors:** Tyson Hillock, James P Smith

**Affiliations:** 1 Surgery, Edward Via College of Osteopathic Medicine, Monroe, USA; 2 General Surgery, St. Francis Hospital, Monroe, USA

**Keywords:** surgical appendectomy, partial cecectomy, ileocecectomy, duplication cyst, appendicular mucocele, right-sided hemicolectomy, appendix cancer, carcinoma of the appendix, mucinous cystadenocarcinoma, surgery general

## Abstract

Low-grade appendiceal mucinous neoplasms (LAMN) constitute a rare subset of epithelial tumors and represent the second most common form of appendiceal cancer. LAMN typically presents as acute appendicitis, with definitive diagnosis often occurring incidentally during appendectomy surgery. While contrast-enhanced computed tomography (CECT) is the imaging of choice, misdiagnoses are common, highlighting the need for additional diagnostic modalities that are often underutilized. There is ongoing debate about treatment recommendations which typically involve a simple appendectomy, but controversy persists regarding the intraoperative necessity of a cecectomy, ileocecectomy, or formal right hemicolectomy. Here, we present a case featuring a 72-year-old African American female referred to our surgery clinic presenting solely with bloating and constipation rather than classical acute appendicitis, discrepancies between CT and MR imaging, and an unusual intraoperative finding of a posteriorly positioned mucocele adhered to the cecum, necessitating right hemicolectomy.

## Introduction

Low-grade appendiceal mucinous neoplasms (LAMN) are rare epithelial cancers, ranking as the second most common tumor of the appendix after neuroendocrine cancers [1]. They predominantly occur in middle-aged females and are often incidentally diagnosed following appendectomy for acute appendicitis, accounting for 0.7-1.7% of all appendix procedures [2-3]. Preoperative imaging modalities such as CT and magnetic resonance imaging (MRI) frequently reveal mucoceles. Definitive diagnosis of LAMN relies on the pathology report following appendectomy [3-4].

LAMN typically manifests with symptoms resembling acute appendicitis due to obstruction of the appendiceal lumen from mucin production. Additional presentations may include bowel obstruction, abdominal distention, intermittent colicky pain, urogenital symptoms, gastrointestinal bleeding associated with intussusception, and weight loss [5-7]. Laboratory findings are inconsistent, although some studies suggest elevated tumor markers such as carcinoembryonic antigen (CEA), cancer antigen (CA) 19-9, and CA-125 may be present. Despite the inconsistency in aiding diagnosis, monitoring these markers has shown beneficial for post-treatment surveillance and recurrence detection [3,7-8].

Common imaging modalities include abdominal ultrasound (US), CT, and MRI, yet distinguishing between non-neoplastic and neoplastic lesions remains inconclusive with reports suggesting approximately 40% of appendiceal mucoceles are originally clinically diagnosed as acute appendicitis [5]. Moreover, reports have demonstrated misdiagnosing ovarian mucinous cysts on imaging to discover appendiceal mucoceles intraoperatively [3-4]. Controversy exists between proper staging and management, prompting recent publications to advocate for a more streamlined and standardized evidence-based approach [4,7].

While current guidelines recommend tumor excision through appendectomy, they also acknowledge disagreements regarding whether to perform a simple appendectomy, cecectomy, ileocecectomy, or a formal right hemicolectomy, often decided intraoperatively at the surgeon’s discretion [5,8]. The prognosis of LAMN is generally favorable, with a 5-year survival rate of 95% when confined to the appendix [7]. However, specific survival rates for different treatment options, such as limited appendectomy or right hemicolectomy with removal or sparing of the ileocecal valve, remain unspecified [2,7-8]. Prognosis is less well-determined when acellular or cellular mucin is found outside the appendix in pseudomyxoma peritonei (PMP), with some suggesting a 5-year survival rate of only 25% [9]. Although a more streamlined treatment approach is generally accepted for PMP, including cytoreductive surgery (CRS) and hyperthermic intraperitoneal chemoperfusion (HIPEC), prophylactic CRS/HIPEC in high-risk patients is heavily debated [2,5,8].

Here, we present a case illustrating a dissimilarity between CT and MR imaging, with an unusual intraoperative finding of a posteriorly placed appendiceal mucocele adhered to the cecum, justifying a formal right hemicolectomy.

## Case presentation

**Figure 1:**
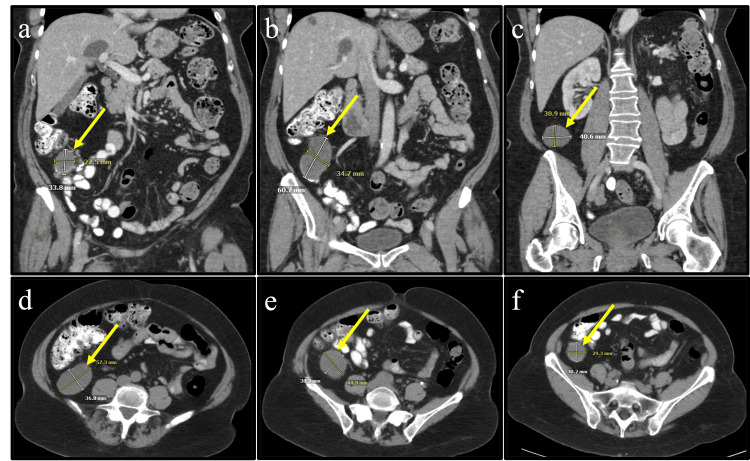
Contrast-enhanced CT imaging highlighting the proposed peritoneal duplication cyst (a) coronal view of the anterior most point of the cystic lesion; (b) coronal view of the middle portion of the cystic lesion; (c) coronal view of the posterior most point of the cystic lesion; (d) transverse view of the superior most point of the cystic lesion; (e) transverse view of the middle portion of the cystic lesion; and (f) transverse view of the inferior portion of the cystic lesion. Note the lack of continuity and absence of noticeable attachment to the appendix or cecum. An absence of contrast within the lesion lumen also suggests separation from the digestive tract. Findings suggest a peritoneal duplication cyst rather than an appendiceal mucocele.

**Figure 2:**
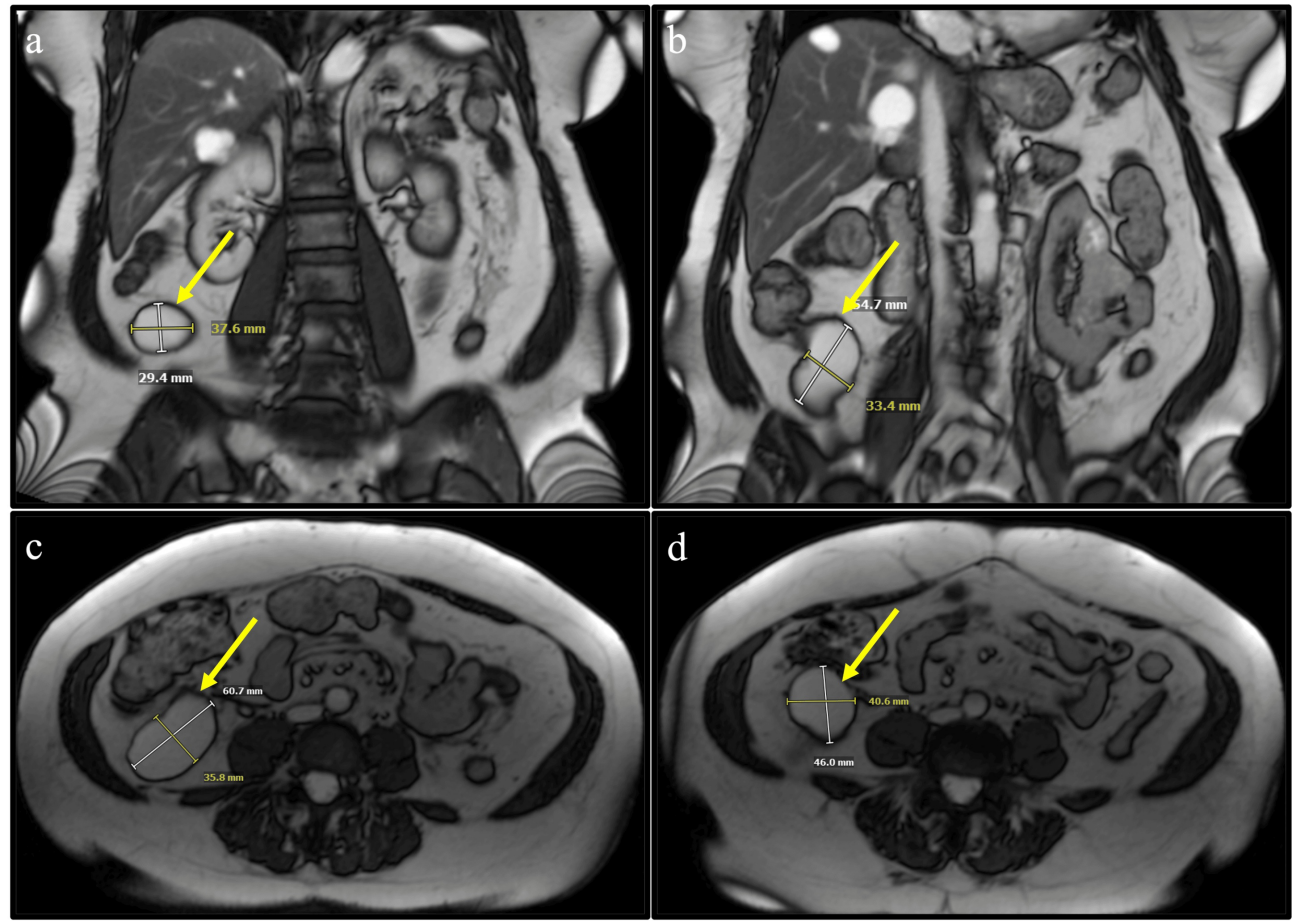
T2-weighted MRI highlighting the proposed appendiceal mucocele (a) coronal view of a posterior point of the cystic lesion; (b) coronal view of an anterior point of the cystic lesion. Note the continuity and attachment of the lesion to the appendix and/or cecum; (c) transverse view of an inferior portion of the cystic lesion; and (d) transverse view of a superior portion of the cystic lesion. Note hyperintense distention of the appendix and bright mucin appearance. Findings suggest appendiceal mucocele rather than peritoneal duplication cyst.

A 72-year-old African American female with a past medical history significant for hypertension treated with amlodipine and losartan, gastroesophageal reflux disease managed with pantoprazole, bilateral cataracts status post bilateral phacoemulsification with intraocular lens implantation, and dysmenorrhea status post partial hysterectomy, was referred to the surgery clinic with complaints of constipation and bloating without abdominal pain. The severity of symptoms gradually increased with longer intervals between bowel movements with significant relief experienced immediately upon defecation. The patient reported intermittent increased use of dietary fiber and milk of magnesia, which provided minimal relief. A screening colonoscopy performed 4 years prior yielded negative results. Initial laboratory investigations, including CBC, CMP, and erythrocyte sedimentation rate (ESR), were unremarkable. Abdominal and pelvic X-rays and US findings were negative. Abdominal CT scan with contrast indicated a cystic mass separate from the appendix, suggestive of a peritoneal duplication cyst (Figure 1). T2 weighted MRI findings contradicted those of the CT scan, suggesting the presence of an appendiceal mucocele (Figure 2). Despite the absence of abdominal pain, fevers, or chills, the patient did report unexpected weight loss and abdominal distention. The assessment included appendiceal mucocele versus peritoneal duplication cyst. It was decided to proceed with an exploratory laparotomy to mitigate the risk of cyst rupture and mass excision with a possible appendectomy or right hemicolectomy depending on intraoperative findings as recommended by guidelines [8].

Intraoperatively, the cystic mass was located posterior to the cecum and appeared to involve a markedly dilated retrocecal appendix measuring 7.9 cm in length with a diameter ranging from 0.7 cm to 3.5 cm. The mass was unusually found to be densely adherent to the posterior edge of the cecum suggesting possible cecal invasion. To prevent rupture and potential seeding of mucin within the abdominal cavity and considering the adherence and suspected invasion of the mass to the cecum, an open right hemicolectomy with division of the ileum approximately 10 cm proximal to the ileocecal valve was performed. The distal ileum, ileocecal valve, cecum, appendix, ascending colon, and proximal transverse colon were excised, along with two periappendiceal and 33 pericolic lymph nodes through high ligation of the ileocolic pedicle. The entire abdomen was surveyed with no signs of PMP or peritoneal metastases. Primary closure of the surgical site ensued. Post-operative recovery was uneventful.

Pathological examination showed dysplastic mucinous epithelial cells diffusely involving the entire 7.9 cm appendix from apex to base, with acellular mucin extending into the mesoappendix, consistent with the diagnosis of LAMN. Margins were negative for tumor, and there was no evidence of lymphovascular or perineural invasion or tumor deposits. Mucin was absent within the right colon and ileum. Additionally, three small tubular adenomas were incidentally discovered in the right colon with no associated mucin. Pathologic stage classification was finalized as pT3 pN0. The patient is scheduled to undergo a repeat abdominal CT scan six months postoperatively, with further surveillance to be determined based on the results.

## Discussion

LAMN typically presents with classical acute appendicitis symptoms, including nonspecific periumbilical pain migrating to the lower right quadrant and signs of peritonitis, attributed to appendiceal distention resulting from tumor growth or mucin production obstructing the lumen [4,7]. However, the disease’s slow progression and varied location on either the appendiceal base or apex can lead to more severe presentations, such as intermittent colicky pain, bowel obstruction, and genitourinary symptoms like oliguria, hydronephrosis, and pyelonephritis due to ureteral obstruction from mass effect [5,7]. In cases of acute obstruction, appendiceal distention can ultimately rupture, leading to the dissemination of mucinous material throughout the abdomen, a condition known as PMP [2,4,10]. Our case illustrates not all patients with LAMN present with acute abdominal pain from appendiceal distension or rupture; instead, symptoms such as abdominal bloating and constipation may predominate leading to the incidental finding of LAMN.

Diagnosing LAMN without surgical excision is near impossible, underscoring the importance of early imaging for detection [7]. Various modalities, including abdominal US, contrast-enhanced CT, and T2-weighted MRI, are recommended. However, as evidenced in our case, discrepancies between imaging modalities may occur, necessitating the need for a comprehensive evaluation [3-4,7]. While the US may reveal a distended cystic appendix with possible porcelain wall calcifications and a lamellated mucinous “onion skin” appearance, CT may depict an enlarged appendix with wall calcifications, thickening, and potential features of PMP, such as septations, calcified nodules, liver scalloping, and thickening of the peritoneum [4,7]. Similarly, MRI may show hyperintense distension of the appendix and mucin appearance on T2-weighted sequences [7]. Frequently, LAMN can present as a cystic mass unrelated to the appendix as seen in our case, often leading to confusion with mucinous ovarian cysts and peritoneal duplication cysts; furthermore, emphasizing the necessity of multimodal imaging before surgical intervention [3-4]. Some propose the role of colonoscopy in diagnosing LAMN, but inconsistencies and the nature of the invasive procedure ultimately dissuade its incorporation into diagnostic practice [3-4,7,10]. If colonoscopy is utilized, one may observe an inflamed appendiceal orifice with potential mucin leaking into the cecal lumen [4]. Our patient last received a colonoscopy only 4 years prior to our surgical intervention at the age of 68 years old, which was negative for polyps, tumors, colonic cancer, or an inflamed mucin-secreting appendiceal orifice. A repeat scope was not recommended at that time due to her age surpassing the upper ends of the United States Preventive Services Task Force (USPSTF) guidelines of 75 years old after the awaited 10-year period [11]. Postoperatively, due to the incidental polyps, our patient is now expected to receive a follow-up colonoscopy as recommended by the USPSTF [11]. It is suggested the progression of LAMN is slow and arguably should have been observed in the endoscopy performed on our patient 4 years prior to diagnosis. However, in our patient’s pathology report, LAMN was confirmed and three incidental colic polyps were discovered regardless of her original negative colonoscopy. This brings to question the possibility of both LAMN and colic polyps progressing quicker than once believed.

While imaging can raise suspicion of LAMN, the decision to proceed with immediate surgical intervention or to observe for a future elective procedure ultimately rests on clinical judgment. A study by Makino et al. proposes the possibility of observative management after reporting their patient did not receive treatment for 12 years without complications. However, their study relies on one account of acute abdominal pain 12 years previously where initial endoscopy and CT imaging were negative but retrospective analysis interpreted an enlarged appendix as an appendiceal mucinous neoplasm. After a therapeutic appendectomy, their patient’s confirmatory LAMN diagnosis was regarded as slowly growing for 12 years [10]. Though difficult to truly say an enlarged appendix retrospectively was a neoplasm, this does pose a question such as how slowly LAMN truly develops. Our case could potentially be recommended within 4 years, while Makino et al. suggest as long as 12.

Early removal of suspicious lesions follows guidelines and is generally well accepted. However, surgical management of LAMN remains controversial, with debates surrounding topics such as performing laparoscopic versus open approaches, and simple appendectomy versus cecectomy, ileocecectomy, or a formal right hemicolectomy [2-3,7-8]. Clinical decisions can be influenced by risk factors of LAMN progression and developing PMP proposed by Guner and Aydin, including high T stage, appendix perforation, presence of acellular mucin on the serosa, and positive surgical margins [5]. Open excision offers benefits to laparoscopy including reduced risk of intraoperative mucocele rupture, development of PMP, and extra-appendiceal recurrence, providing comfort to the surgeon; though, no true comparative studies are reported [3,7]. For localized lesions, complete removal of the appendix with a portion of the cecum is often recommended [7,10]. However, the lack of staging and clear surgical margins until post-appendectomy justifies caution. Intraoperative frozen sections are discouraged due to the high pathologic complexity of these lesions, potentially leading to reoperation if margins remain positive; therefore, some argue utilizing the right hemicolectomies to diminish the risk [7,10]. Current recommendations advise preserving the ileocecal valve [7]. However, given that LAMN most commonly manifests at the base of the appendix, some surgeons choose to excise the distal ileum to mitigate the risk of subsequent reoperation [2,10]. Ultimately, the choice of surgical approach and extent of organ resection is contingent upon the surgeon’s clinical judgment and experience [8]. In our case, the decision to perform an open right hemicolectomy with ileocecal valve removal was motivated by potential mucocele rupture, tumor adherence to the posterior cecum, and the absence of clear intraoperative margins, ultimately alleviating the risk of neoplastic spread into adjacent structures and subsequent reoperations. Our decision was further justified by pathology showing tumor invading through the muscularis propria into the subserosa with acellular mucin in the mesoappendix (pT3 score).

Interestingly, a case series by Guner and Aydin suggests that only 60.3% of LAMN patients present in emergent conditions, with the remaining 39.6% undergoing elective surgeries [5]. Arguably, this allows for nearly 40% of cases where conservative management may be warranted when LAMN is suspected. Wang et al. discuss five cases in which three of their patients opted for delayed surgery anywhere from two to five months, with the condition that close monitoring would be performed for progression. The delayed intervention had little to no repercussions [7]. These findings, along with those of Makino et al. [10], suggest the possibility of conducting a complete workup and tumor staging prior to surgical intervention for select patients, thereby providing clear guidance for decision-making regarding a simple appendectomy, cecectomy, ileocecectomy, or a formal right hemicolectomy.

## Conclusions

Our case highlights the challenges posed by discrepancies between CT and MR imaging, leading to uncertainty regarding a peritoneal duplication cyst or an appendiceal mucocele. An appendiceal mucocele should raise suspicion of LAMN or other mucinous neoplasms. Surgical intervention of LAMN is based on the surgeon’s intraoperative discretion due to the final pathology report acquired postoperatively and possible reoperation. The adherence of our patient’s neoplasm to the posterior cecum and the risk of mucocele rupture warranted the intraoperative decision to perform an open right hemicolectomy, further justified postoperatively with acellular mucous present in the mesoappendix.
